# Respiratory Syncytial Virus-Load Kinetics and Clinical Course of Acute Bronchiolitis in Hospitalized Infants: Interim Results and Review of the Literature

**DOI:** 10.3390/pathogens12050645

**Published:** 2023-04-27

**Authors:** Giulia Piccirilli, Alessandro Rocca, Eva Caterina Borgatti, Liliana Gabrielli, Daniele Zama, Luca Pierantoni, Marta Leone, Camilla Totaro, Matteo Pavoni, Tiziana Lazzarotto, Marcello Lanari

**Affiliations:** 1 Microbiology Unit, IRCCS Azienda Ospedaliero-Universitaria di Bologna, Via Massarenti 9, 40138 Bologna, Italy; 2 Pediatric Emergency Unit, IRCCS Azienda Ospedaliero-Universitaria di Bologna, Via Massarenti 9, 40138 Bologna, Italy; 3 Department of Medical and Surgical Sciences (DIMEC), University of Bologna, 40138 Bologna, Italy; 4 Specialty School of Pediatrics, Alma Mater Studiorum, University of Bologna, 40126 Bologna, Italy

**Keywords:** respiratory syncytial virus, acute bronchiolitis, hospitalized infant, viral load, viral kinetics, oxygen therapy, length of stay in hospital, clinical score

## Abstract

Respiratory Syncytial Virus (RSV) bronchiolitis is the leading cause of hospitalization in infants. The role of RSV load in disease severity is still debated. We present the interim results of a prospective monocentric study enrolling previously healthy infants hospitalized for RSV bronchiolitis, collecting nasopharyngeal aspirates every 48 h from admission to discharge, and evaluating RSV load dynamics in relation to clinical outcome measures of bronchiolitis severity, including: need, type and duration of oxygen therapy, length of hospitalization, and the bronchiolitis clinical score calculated at admission. The results showed that the highest viral replication occurs within the first 48 hours after admission, with a significant decrease at subsequent time points (*p* < 0.0001). Moreover, higher RSV-RNA values were associated with the need for oxygen therapy (*p* = 0.03), particularly high-flow nasal cannula type (*p* = 0.04), and longer duration of respiratory support (*p* = 0.04). Finally, higher RSV load values were correlated with lower white blood cells, especially lymphocyte counts and C-reactive protein levels (*p* = 0.03, *p* = 0.04, and *p* = 0.01, respectively), as well as with patients of a younger age (*p* = 0.02). These data suggest that RSV may actively contribute to the clinical severity of bronchiolitis, together with other potential non-viral factors.

## 1. Introduction

Respiratory syncytial virus (RSV) is an enveloped, negative-sense single-stranded RNA virus that belongs to the *Pneumoviridae* family, genus *Orthopneumovirus* [1]. It represents the main infectious causative agent of acute bronchiolitis (up to 80% of cases) [2], characterized by an inflammatory process with edema, increased mucus production, and necrosis of the epithelial cells lining the small airways that can lead to hospital admissions in infants [3]. Treatment of acute viral bronchiolitis is mainly supportive (respiratory and fluid management) and is currently without a specific antiviral therapy [4]. To date, there is no approved vaccine against RSV, so that environmental prophylaxis strategies remain crucial in preventing and limiting the spread of infection. Pharmacological immunoprophylaxis, involving palivizumab and the more recent nirsevimab, is still indicated mostly for the prevention of serious RSV lower-respiratory-tract disease requiring hospitalization [5]. The main known risk factors for a severe clinical course are related to host comorbidities, such as congenital heart or chronic pulmonary diseases and prematurity [4,6]. However, considering that many patients without comorbidities also present a severe course, other aspects, including viral factors and immune-inflammatory host response, are thought to play a role in the variability of the clinical manifestations [7]. In particular, the role of RSV load as a pathogen-related risk factor is debated. Such great variability in the results obtained from different studies could be related to the analysis of RSV load in relation to diverse outcomes evaluated as markers of bronchiolitis severity: need and duration of oxygen therapy, mechanical ventilation, admission to a pediatric intensive care unit (PICU), hospital length-of-stay (LOS), or clinical scores of severity [7,8,9,10,11,12,13,14,15,16,17,18,19,20,21,22,23,24,25,26]. Moreover, many studies based their analyses on the results obtained from a single sample for each included patient, losing the possibility of evaluating viral-replication kinetics [9,10,11,12,13,15,16,18,19,20,21,22,23,25].

Aware of this great variability in outcomes and results, we chose to conduct a study including the largest part of bronchiolitis severity markers evaluated by other authors. In this study, the association between RSV load, the kinetics of viral replication, and the clinical course of RSV bronchiolitis in hospitalized, previously healthy infants was investigated, to define a direct role of viral replication in the severity of the disease. The results are presented with a complete literature review.

## 2. Materials and Methods

This is an ongoing prospective observational monocentric study conducted in the Pediatric Emergency Unit and the Laboratory of Virology, Microbiology Unit, of the IRCCS Policlinico di Sant’Orsola, Azienda Ospedaliero-Universitaria di Bologna, Italy. Infants aged one year or younger hospitalized in the Pediatric Emergency Unit for a first episode of acute bronchiolitis between March 2019 to November 2021 and whose parents gave consent for participation in the study were included for this preliminary analysis. Of note, this period of enrollment included the so-called “first wave” of the severe acute respiratory syndrome coronavirus 2 (SARS-CoV-2) pandemic. Children affected by chronic conditions known to be host risk factors for severe RSV infection (chronic cardiopulmonary or neuromuscular disease, cystic fibrosis, congenital or acquired immunodeficiencies) or by other concurrent breathing diseases causing respiratory distress were excluded. Bronchiolitis was defined as the presence of a history of upper -respiratory-tract infection followed by the acute onset of respiratory distress with a cough, tachypnea, retractions, and diffuse crackles on auscultation, according to the Italian inter-society consensus for acute bronchiolitis [6].

At enrollment, for all subjects, demographic and clinical data (age, gender, ethnicity, weight on admission, breastfeeding and premature birth, duration of symptoms prior to hospitalization), as well as laboratory findings (total and differential white blood-cell counts—WBC, and C-reactive protein—CRP values), were collected.

Different outcomes were considered as markers of disease severity, such as the need, duration, and type of oxygen therapy, distinguishing among low-flow and high-flow nasal cannulae (HFNC) support, PICU admission, hospital LOS and bronchiolitis clinical score (BCS). The BCS was selected from the literature and calculated for each patient on admission, ranging from 0 to 12 points according to the respiratory rate (0: <50/min, 1: 50–60/min, 2: 61–69/min, 3: ≥70/min), direct respiratory distress signs (0: absent, 1: intercostal retractions, 2: intercostal retractions and nasal flaring, 3: intercostal retractions, jugular retractions, and nasal flaring), indirect respiratory distress signs (0: normal feeding, 1: reduced feeding, 2: reduced feeding and irritability, 3: reduced feeding and lethargy), auscultation (0: normal, 1: expiratory wheezing or crackles, 2: expiratory wheezing and crackles, 3: inspiratory and expiratory wheezing and/or reduction in murmur). According to BCS, the severity grading of bronchiolitis was stratified into 3 groups: mild (1–4 points), moderate (5–8 points), and severe (9–12 points) [27].

For each patient, nasopharyngeal aspirates (NPA) were collected at different time points, in particular upon admission (day 0, d0) and every 48 h during hospitalization until discharge (d2, d4, d6, d8, and so on). On the NPA collected at admission, the direct diagnosis of respiratory viral infection was performed by the laboratory of virology. In particular, the antigens of the eight most common respiratory viruses were detected by direct immunofluorescence assays: RSV, influenza virus A and B, parainfluenza virus 1–3, and adenovirus were identified by the SimulFluor^®^ Respiratory Screening kit (light diagnostics™ Chemicon Internacional, Temecula, CA, USA), while metapneumovirus was detected by human metapneumovirus direct immunofluorescence assay (light diagnostics™). In addition, since the spreading of the coronavirus disease 2019 (COVID-19) pandemic, according to our hospital procedures, universal pre-admission screening of SARS-CoV-2 testing during hospitalization was performed by molecular methods; no positive cases were observed. All the NPA samples were stored at −80 °C. For this preliminary analysis, NPA that resulted positive only for RSV were retrospectively analyzed for viral-load quantification. Briefly, nucleic acid extraction was performed by NucliSENS^®^ easyMag^®^ instrument (bioMérieux, Marcy-l’Étoile, France), according to the manufacturer’s instructions (initial volume of 200 µL of each NPA eluted in 50 μL). The reverse transcription and quantitative amplification of the viral target (gene matrix) were performed by a home-made quantitative real-time TaqMan PCR (RT-qPCR). The reaction was carried out at a total volume of 10 µL containing 4 µL of 2.5× Universal Master Mix (DiaSorin, Saluggia, Italy), 0.5 µL of Reverse Transcriptase (DiaSorin, Saluggia, Italy), 0.2 µL of control primer pair (SimplexaTM Extraction and Amplification Control Set-RNA, DiaSorin), 0.2 µL of RSV (Respiratory Syncytial Virus) primer pair (DiaSorin, Saluggia, Italy), 0.1 µL of RNAse free water and 5 µL of the extracted sample. Thermocycling conditions were performed on LIAISON^®^ MDX (DiaSorin, Saluggia, Italy) in a 96-well format as follows: 50 °C for 8 min, 97 °C for 2 min, followed by 40 cycles of 97 °C for 10 s, 56 °C for 30 s (data collection). A synthetic oligonucleotide (such as gBlocks^®^ Gene Fragments, Tema Ricerca, Castenaso, Italy), 770 bp in length, of the matrix gene, was used to perform ten-fold dilutions and generate a standard curve. Before RT-qPCR, human DNA quantification in each eluted sample was performed using NanoDrop™ 2000 spectrophotometer (Thermo Fisher Scientific, Waltham, MA, USA). The RSV viral load was reported as log_10_ copies/ng human [h]DNA. For values under the lower limit of quantification (LLoQ) an arbitrary value of half of the LLoQ was assigned (1.6 log_10_ copies/ng hDNA), while negative samples were reported as 1 log_10_ copy/ng hDNA.

Categorical variables were presented as frequencies. Continuous variables were presented as median (interquartile range, IQR) or mean ± standard deviation (SD), according to data distribution, verified for each variable by the Kolmogorov–Smirnov test. Data were compared using Χ^2^, Mann Whitney, Student t, or Kruskal–Wallis test, as needed, based on the Kolmogorov-Smirnov test. Correlation analysis was performed by Spearman test. Statistical analyses were conducted using GraphPad Prism 8.0.1 version (GraphPad Software, Inc., San Diego, CA, USA) and a *p* value < 0.05 was considered statistically significant.

This study was approved by the Area Vasta Emilia Centro Ethics Committee (reference 737/2018/Sper/AOUBo).

## 3. Results

### 3.1. Analysis of Study Patients’ Demographic and Clinical Characteristics 

For this preliminary analysis, a total of 38 patients with RSV bronchiolitis were analyzed during the hospitalization period (50% males). Infants presented a mean age of 3.9 ± 3.0 months and a mean weight of 5.9 ± 1.8 Kg on admission. The study population included predominantly full-term subjects, with a mean gestational age at birth of 38.8 ± 2.3 weeks; six (15.8%) infants were preterm babies (range 32–36 weeks of gestational age). Thirty-two (84.2%) were being breastfed upon admission, and none were exposed to passive smoke at home. The mean duration of symptoms prior to admission was 3.5 ± 2.2 days. Upon hospitalization, the subjects had a mean value of 36.8 ± 0.9 °C for body temperature, 52.3 ±11.4 breaths per minute for respiratory rate, 155.2 ± 20.0 beats per minute for heart rate, and 95.6 ± 2.9% for oxygen peripheral saturation (SpO_2_). The mean BCS on admission was 4.0 ± 1.5 points. As described in the previous section, patients were stratified into two BCS severity groups: 30 (78.9%) and eight (21.1%) patients with mild and moderate bronchiolitis, respectively. No infants had severe BCS. Twenty-four (63.2%) infants required respiratory support with oxygen therapy, five (20.8%) of them with HFNC, and the remaining 19 (79.2%) received standard low-flow support. The global mean duration of oxygen therapy was 88.3 ± 44.0 h, with 13 (54.2%) patients needing this support for ≤72 h. The overall median hospital LOS was five days (IQR = 5–7). No patient died, nor did they need non-invasive or invasive ventilatory support, nor admission to PICU during hospitalization.

### 3.2. Analysis of RSV Load and Kinetics of Viral Replication during Hospitalization Independently by Bronchiolitis Clinical Course 

**Figure 1:**
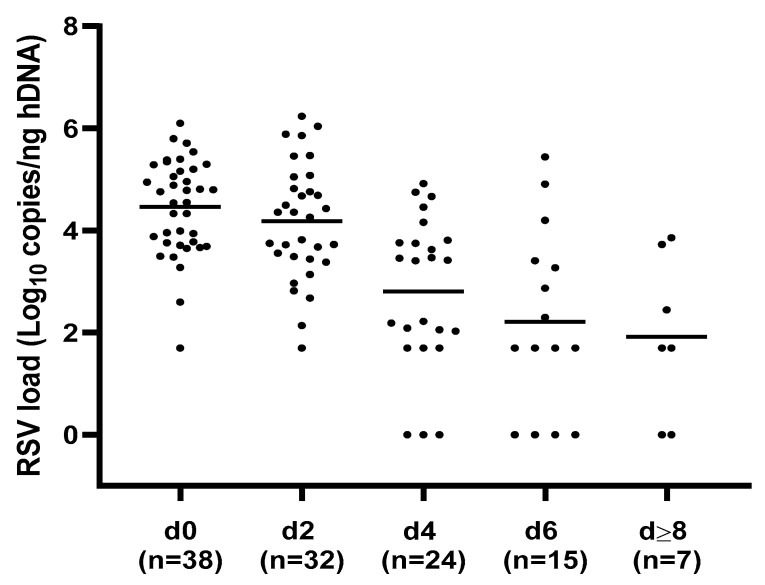
Respiratory syncytial virus (RSV) load values distribution at different time points. The highest RSV load mean values were detected within 48 hours of admission, with a significant decrease in the following time points; *p* < 0.0001. h = human, d = day, d0 = admission, n = number of NPA samples analyzed for each time point.

A total of 116 respiratory samples were collected from the 38 hospitalized patients with RSV infection and analyzed for viral-load quantification. A preliminary assessment of RSV load kinetics was performed regardless of the clinical course of the bronchiolitis. In particular, a median of three NPA for each case (range = 2–6) according to the duration of hospitalization, was collected and evaluated. The highest RSV load mean values were detected within 48 h of admission (d0: 4.46 ± 0.94; d2: 4.18 ± 1.13 log_10_ copies/ng hDNA) with a significant decrease in the following time points (d4: 2.8 ± 1.94 to d ≥ 8: 1.92 ± 1.57; *p* < 0.0001), as shown in Figure 1.

**Figure 2:**
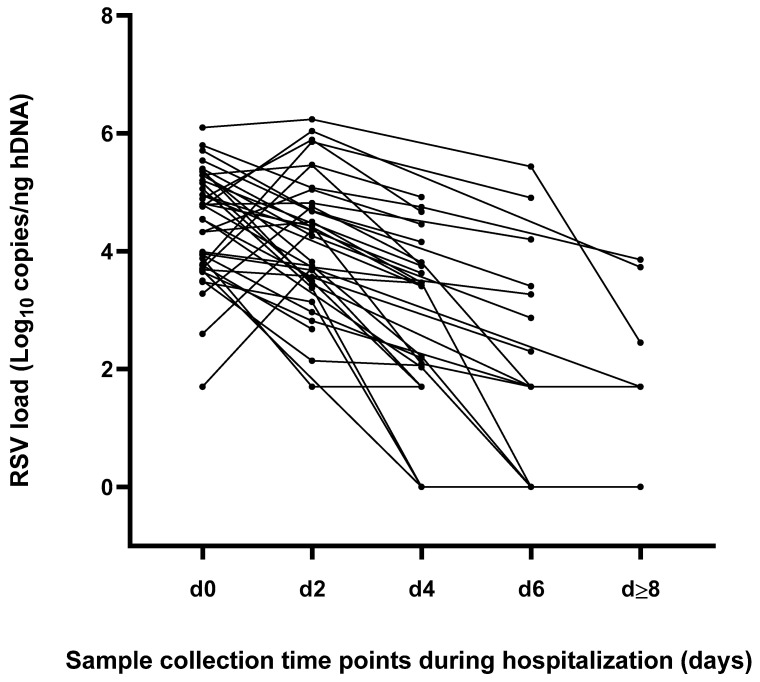
Kinetics of viral replication represented as individual RSV load at different time points during the hospitalization period.

As reported in Figure 2, the peak of RSV load was documented in 26 (68.4%) cases upon admission (d0), while in the remaining 12 (31.6%) patients it was reached after 48 h from hospitalization (d2). A consistent viral clearance at the discharge time point was observed in 14 (36.8%) patients, who resulted completely negative (six cases, 42.9%) or positive, with a viral load under the LLoQ (8 cases, 57.1%); these infants presented a median hospital LOS equal to five days (IQR = 5–7). Of note, for three patients RSV-RNA negative results were observed both upon discharge and at previous time points.

**Figure 3:**
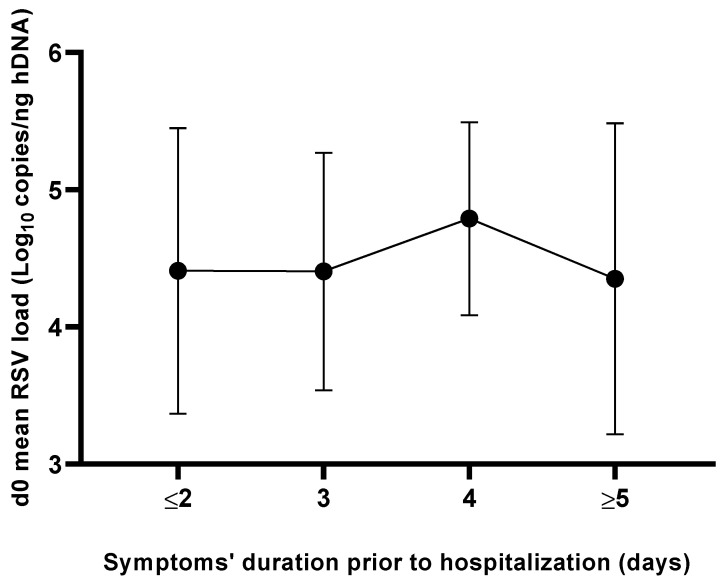
Mean RSV load levels at admission (d0) as a function of duration of symptoms prior to hospitalization.

The overall mean time of symptom onset prior to hospitalization was equal to 3.5 ± 2.2 days and no statistically significant difference (*p* = 0.87) was observed when comparing the mean RSV load values detected at the d0 time point stratified according to the beginning of symptoms (Figure 3).

### 3.3. Impact of RSV Load and Non-Viral Risk Factors (Patients’ Characteristics and Laboratory Findings) on the Disease Severity

As first markers of disease severity, the need, type, and duration of oxygen therapy were analyzed in relation to RSV load and non-viral risk factors such as demographic, andclinical patients’ characteristics, as well as laboratory findings. The results are summarized in Table 1.

**Figure 4:**
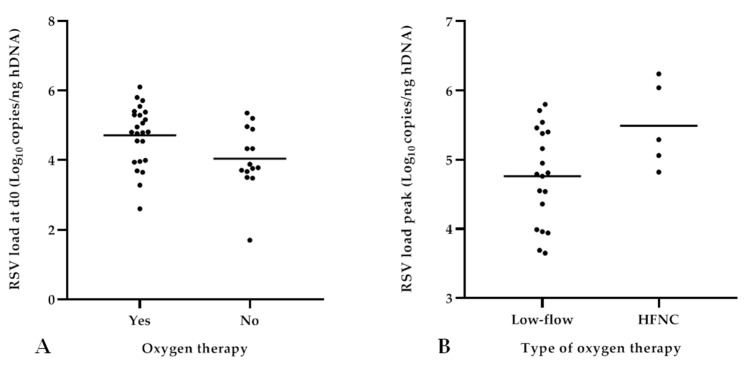
Analysis of RSV load in relation to need and type of oxygen therapy. Higher viral loads detected at admission (d0) in patients needing oxygen therapy (*p* = 0.03) (**A**); higher RSV load peak values reached within 48 h from admission detected in patients treated with HFNC (*p* = 0.04) (**B**).

Concerning RSV load analysis at different time points, significantly higher RSV-RNA levels were detected at admission (d0) in patients needing oxygen therapy (mean values 4.71 ± 0.87 vs. 4.04 ± 0.93; *p* = 0.03) compared to those without supplemental oxygen (Figure 4a). In particular, patients receiving HFNC therapy reached a higher RSV load peak value compared to those treated by low-flow oxygen therapy (5.49 ± 0.62 vs. 4.76 ± 0.69, respectively; *p* = 0.04), as shown in Figure 4b. Specifically, among the cases treated with HFNC, 60% (3/5) achieved the RSV load peak 48 h after admission; albeit without statistical significance, the mean RSV load measured at d2 was higher in infants receiving HFNC compared to those receiving low-flow oxygen therapy (4.95 ± 1.20 vs. 4.06 ± 0.78, respectively; *p* = 0.07). Consequently, patients who needed HFNC presented a reduced, albeit not significant, viral decay (∆ d0–d2) compared to those who required low-flow oxygen therapy. No significant differences in terms of need, type, and duration of oxygen therapy emerged regarding both the RSV load detected at subsequent time points after admission (≥d2) as well as the viral decay.

When analyzing the same outcomes in relation to non-viral risk factors, no differences emerged regarding the demographic features, except for ethnicity, as Caucasian patients required oxygen therapy more frequently (*p* = 0.04). Of note, the extremely low frequency of other races in our study population could have affected this result. Neither clinical, including prematurity, weight on admission, and breastfeeding, nor laboratory characteristics, namely complete and differential leukocyte cell counts and CRP values, seemed to impact disease severity when considering oxygen therapy, with the exception of neutrophil cell count, that resulted higher, albeit without statistical significance, in patients receiving HFNC compared to those treated with low-flow oxygen therapy (6,419 [4,928–6,926] vs. 3,510 [2,245–5,272]; *p* = 0.06). The duration of symptoms before hospitalization was not correlated with the duration of oxygen therapy.

Hospital LOS and BCS at admission were analyzed as additional markers of disease severity in relation to RSV load and non-viral risk factors, such as demographic and clinical patients’ characteristics, as well as laboratory findings; results are shown in Table 2. No differences were identified when analyzing RSV-RNA levels at different time points in relation to these outcomes.

Considering non-viral risk factors, younger patients had a significantly longer hospitalization (mean age 4.8 ± 3.2 vs. 2.6 ± 2.1 months; *p* = 0.03). Moreover, infants with a greater hospital LOS showed higher monocyte cell count values (836 ± 322 vs. 1,203 ± 432 × 10^9^ cell/L; *p* = 0.01). Concerning BCS at admission, a greater basophil cell count was observed in cases classified as moderate compared to mild (median values: 29 [22–43] vs. 52 [38–58], respectively; *p* = 0.04). No other differences were identified considering the remaining patients’ demographic and clinical variables, or their laboratory findings.

In addition, a correlation analysis between hospital LOS and patients’ variables (demographic characteristics, clinical features), laboratory findings, and clinical outcomes considering only continuous data was also evaluated (Spearman test). A negative correlation emerged between the patient’s age and hospital LOS (r −0.38, *p* = 0.02), confirming the evidence obtained by dichotomizing the patients into two groups (LOS ≤ 5 or > 5 days, Table 2): younger patients presented a longer LOS (*p* = 0.03). The same inverse correlation, trending to significance, was observed when considering LOS and patient weight on admission (r −0.31, *p* = 0.05).

Based on the obtained data concerning viral load, results for RSV-RNA levels detected at d0 and at the peak values reached within 48 h after admission were analyzed in correlation with all continuous reported variables (patients’ demographic and clinical characteristics, laboratory findings, duration of oxygen therapy and hospital LOS as clinical outcomes). The results are summarized in Table 3.

**Figure 5:**
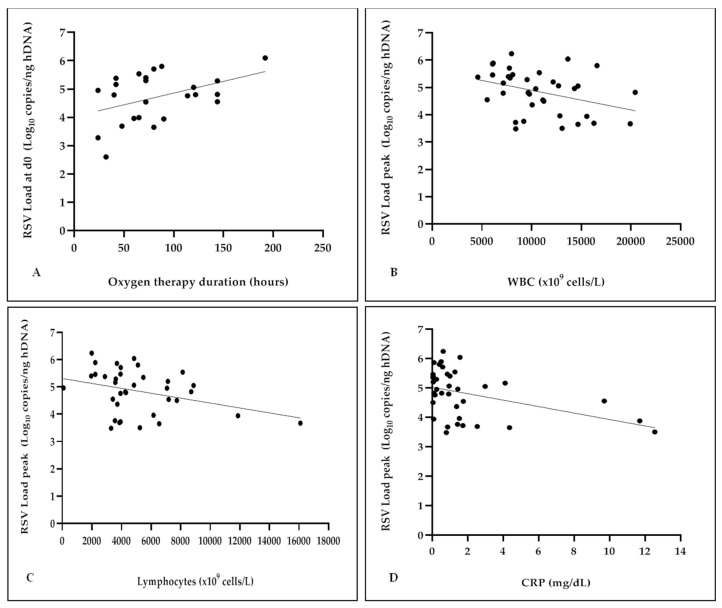
Analysis of RSV load in correlation with duration of oxygen therapy, the total WBC and the lymphocyte cell count as well as the CRP values. Positive correlation between the RSV-RNA levels at d0 and the duration of oxygen therapy (r 0.42, *p* = 0.04) (**A**); negative correlation between the RSV load peak and total WBC (r −0.35, *p* = 0.03) (**B**); negative correlation between the RSV load peak and the lymphocyte cell count (r −0.34, *p* = 0.04) (**C**); negative correlation between the RSV load peak and the CRP values (r −0.40, *p* = 0.01) (**D**).

As detailed in Figure 5a, a positive correlation between the RSV-RNA levels at d0 and the duration of oxygen therapy was found (r 0.42, *p* = 0.04), although no significant differences emerged when patients were dichotomized on 72 h cut-off (as shown in Table 1), which was chosen by considering the mean time duration of oxygen therapy. On the contrary, a negative correlation was observed when evaluating the RSV load peak in association with the total WBC and the lymphocyte cell count as well as the CRP values (r −0.35, *p* = 0.03; r −0.34, *p* = 0.04; r −0.40, *p* = 0.01, respectively), as shown in Figure 5b–d. 

## 4. Discussion

RSV infection may cause severe bronchiolitis in infants, leading to hospitalization, PICU admission, and potentially death [7,28,29]. 

The direct role of RSV on the severity of bronchiolitis clinical course is debated because of the different results obtained in previous studies by other authors, who have reported direct, inverse, or absent relationships between higher viral loads and the severity of the disease (see Supplementary Tables S1–S3) [7,8,9,10,11,12,13,14,15,16,17,18,19,20,21,22,23,24,25,26]. 

The diversity of the results could be linked to differences in: (*i*) the type of study design (e.g. prospective vs. cross-sectional); (*ii*) enrollment criteria (e.g. subjects’ age, outpatients and/or hospitalized patients with comorbidities or drug-exposure inclusion); (*iii*) type of specimens; (*iv*) quantification methods of RSV load (e.g. culture titers vs. molecular technologies); and (*v*) parameters considered to evaluate the severity of the clinical course.

To our knowledge, this is the first study that evaluates the RSV load kinetics normalized on the amount of hDNA in the NPA samples collected from only hospitalized previously healthy children with acute bronchiolitis, all under one year of age, according to the European strict definition of bronchiolitis [6].

In addition, we tried to limit the above-mentioned great variability by including the largest part of bronchiolitis severity markers evaluated by other authors. 

A preliminary assessment of RSV load kinetics was performed, regardless of the clinical course of bronchiolitis, in order to evaluate the dynamics of viral replication during the infectious episode until discharge. The results showed that the highest viral replication occurs within 48 h after admission, with a significant decrease at the following time points (*p* < 0.0001). These results are in agreement with those in the literature [7,11,14,17,24,26]. 

Some authors found that RSV load at admission is affected by the duration of symptoms before hospitalization [11]. In our study, no statistically significant differences were found stratifying RSV load according to the onset of symptoms prior to hospitalization. 

In the second part of the study, the impact of viral replication on the clinical course of bronchiolitis was analyzed considering the need, type, and duration of oxygen therapy as measures of disease severity. Comparing patients who needed oxygen therapy with those who did not, our data showed higher RSV load values during the first 48 hours after hospitalization, with a statistical significance upon admission (*p* = 0.03). In addition, we found a positive association between RSV loads and the duration of oxygen therapy when this factor was considered as a continuous variable (*p* = 0.04). These findings confirmed the evidence reported by other authors [19,25]. When analyzing the type of oxygen therapy, patients who needed HFNC support presented higher RSV-RNA levels within 48 h after hospitalization, and significant differences were observed when peak values were considered (*p* = 0.04). HFNC therapy can be considered as a rescue respiratory treatment for children not adequately supported by standard low-flow oxygen therapy before switching them to non-invasive or invasive ventilation [30]. Consequently, as no patient required PICU admission, an outcome included in our study, the need for HFNC was considered as the expression of a great clinical respiratory effort and bronchiolitis severity. The results regarding the RSV load of infants who needed oxygen therapy, and in particular HFNC (evaluated for the first time, to our knowledge) seemed to corroborate studies where the findings underlined an association between higher RSV load and the need for respiratory support [11,12,19]. In addition, in our study, the patients treated by HFNC also had a reduced, albeit not significant, viral decay (∆ d0–d2) compared to those who required low-flow oxygen therapy. Of note, in the present study, only five patients received HFNC; no other association emerged when RSV load values were analyzed in relation to other clinical outcomes, such as hospital LOS and BCS at admission, which was similar to observations reported by other authors [10,15,20,26].

Some studies reached conclusions in contrast to ours. These differences could be due to the inclusion of diverse study populations, such as outpatients or those admitted to PICU, or to the variability on the parameters included in the adopted clinical scores [7,13,16,17,22]. Future investigations will be extended to a larger cohort in order to better define if there are differences in viral replication kinetics in relation to the clinical course of RSV bronchiolitis; this would probably also allow for the inclusion of patients in PICU.

The impact of non-viral risk factors was also investigated in this study in terms of demographic and clinical variables and laboratory parameters expressing the immune and inflammatory host response to RSV infection. A statistically significant difference was highlighted for ethnicity in infants needing oxygen therapy. However, the extremely low frequency of non-Caucasian race subjects in our study population could have affected this result, although the same findings were reported by other authors [7,10,11,14,18]. Wright et al. hypothesized a different ethnicity-associated immune-inflammatory response [10]. 

Analyzing patients’ age upon admission, our results showed a significant correlation with hospital LOS (*p* = 0.03), showing that younger infants required a longer period of hospitalization. A consistent number of studies have reported that the clinical course is affected by the age of patients, with younger children being at risk for a more severe illness [10,11,12,15,16,24,25]. Finally, in our analyses, an inverse correlation was found between the peak RSV load and total WBC (*p* = 0.03), lymphocyte counts (*p* = 0.04), and CRP values (*p* = 0.01). These results could reflect a reduced ability to control viral replication. Alternatively, the decrease in WBC and lymphocyte counts in the peripheral blood could be the consequence of a more severe infection, eliciting cell migration from blood to the lung tissue. RSV infection may finally activate other inflammatory mediators, such as cytokines or chemokines, that we have not investigated until now.

In conclusion, our interim results showed a direct association between higher RSV-RNA levels within the first 48 hours after admission and a greater need for oxygen therapy, particularly the HFNC type, and a longer duration of respiratory support. However, non-viral risk factors (such as patient’s age and inflammatory/immune response) probably also contribute to the clinical severity of bronchiolitis during RSV infection in infants. 

These results represent the preliminary data of a study that included a larger population size enrolled from March 2019 and is still ongoing. Here, we evaluated the first patients included up to November 2021, thus considering the so-called “first wave” of the COVID-19 pandemic. Although no significant differences in RSV clinical manifestations were observed, the spreading of SARS-CoV-2 impacted both the seasonality of the spread of RSV [31,32] and the age of infected children [33]. In particular, throughout the 2020–2021 epidemic season, the adopted restrictions to limit the spread of SARS-CoV-2 led to a rapid decline of RSV circulation and a significant decrease in hospitalizations for bronchiolitis at our center [34]. Conversely, in the following epidemic season 2021–2022, an unseasonal RSV circulation with a peak in early November 2021 was reported in Italy and the median age of RSV-infected children was higher than that observed in the same period prior to the COVID-19 pandemic [32,33]. In fact, children between two and three years of age resulted more affected by RSV than younger infants. All the aforementioned factors impacted our study enrolment [33].

This preliminary analysis, considering the first enrolled patients, presents many strengths: (*i*) RSV load was normalized on the amount of human DNA, providing a reduced intrinsic variability in the sample collection, (*ii*) sequential sampling was used to study the kinetics of viral replication during hospitalization, (*iii*) the study included a selected and homogeneous population of previously healthy infants within one year of age, (*iv*) the largest part of disease severity markers was included.

In addition, in contrast with other studies, we enrolled only hospitalized infants based on several clinical factors, such as respiratory distress and the need for oxygen therapy, but also elements considered in BCS, such as lethargy or irritability, that can underline severe infection, or reduced feeding, with the need for enteral or parenteral nutrition or hydration. Infants with social risk factors, such as parental reliability or distance from the hospital, were not enrolled in the study because they remained, according to our hospital procedures, in the Pediatric Emergency Department-Intensive Brief Observation area.

This study also has some limitations, such as the small sample size and the lack of infants who required PICU admission. Moreover, we did not study RSV serotypes and genotypes, nor did we evaluate the presence of pathogens other than the most common respiratory viruses. Our analyses need to be extended by increasing the number of subjects already enrolled in the ongoing study, thereby possibly including infants that require PICU admission and exploring host responses to viral infection by evaluating lymphocyte subtype cells and their derived cytokines in order to obtain a better comprehension of the mechanisms that lead to greater severity in this very common disease.

## Figures and Tables

**Table 1:** Need, type and duration of oxygen therapy in relation to demographic and clinical patients’ characteristics, laboratory findings and respiratory syncytial virus (RSV) load (mean log_10_ copies/ng hDNA). Continuous variables were presented as median [IQR] or mean ± SD, according to data distribution. n.e.^§^: statistic test not evaluable (frequency data equal to 0); n.e.^$^: data not evaluable (≤1 available data for row or column); *: data not available for all subjects; d = day, d0 = admission; ∆: viral decay; significant correlations are highlighted in bold type.

	Need of Oxygen Therapy			Type of Oxygen Therapy			Duration of Oxygen Therapy (hours)		
	Yes (n = 24)	No (n = 14)	*p* Value	HFNC (n = 5)	Low-Flow (n = 19)	*p* Value	≤72 h (n = 13)	>72 h (n = 11)	*p* Value
**RSV load**									
d0	4.71 ± 0.87	4.04 ± 0.93	**0.03**	5.20 ± 0.55	4.58 ± 0.90	0.16	4.51 ± 0.93	4.95 ± 0.75	0.22
d2	4.28 ± 0.95	4.03 ± 1.41	0.55	4.95 ± 1.20	4.06 ± 0.78	0.07	4.09 ± 1.22	4.47 ± 1.17	0.39
d4	2.82 ± 1.24	2.78 ± 1.92	0.95	n.e.^$^	n.e.^$^	n.e.^$^	3.01 ± 0.88	2.54 ± 1.71	0.49
d6	2.36 ± 1.59	2.46 ± 3.47	0.95	n.e.^$^	n.e.^$^	n.e.^$^	2.06 ± 1.25	2.27 ± 2.04	0.83
Peak	4.91 ± 0.73	4.59 ± 0.90	0.24	5.49 ± 0.62	4.76 ± 0.69	**0.04**	4.77 ± 0.62	5.08 ± 0.84	0.30
∆ d0–d2	0.44 ± 1.02	−0.06 ± 1.46	0.26	0.25 ± 1.14	0.51 ± 1.01	0.64	0.37 ± 1.22	0.52 ± 0.83	0.75
∆ d0–d4	1.73 ± 1.50	1.33 ± 1.62	0.55	n.e.^$^	n.e.^$^	n.e.^$^	n.e.^$^	n.e.^$^	n.e.^$^
**Demographic characteristics**									
Age (month)	3.4 ± 2.9	4.8 ± 2.9	0.18	3.7 ± 2.9	3.4 ± 3.0	0.83	4.1 ± 3.4	2.7 ± 2.2	0.27
Sex (male)	12	7	1.00	3	9	1.00	8	4	0.41
Ethnicity (total available n *)	(n * = 22)	(n = 14)	**0.04**	(n = 5)	(n * = 17)	n.e.^§^	(n * = 11)	(n = 11)	n.e.^§^
Caucasian	20	8		5	15		10	10	
Asian	1	1		0	1		0	1	
African-American	1	5		0	1		1	0	
**Clinical characteristics**									
Premature birth (<37 WG)	3	3	0.53	1	2	0.32	1	2	0.59
Weight on admission (kg)	5.6 ± 1.7	6.5 ± 1.8	0.17	6.0 ± 1.6	5.4 ± 1.8	0.52	5.9 ± 2.0	5.1 ± 1.4	0.29
Breastfeeding	19	13	0.26	3	16	0.27	12	7	0.14
Duration of symptoms prior to d0 (days)	3.5 ± 2.2	3.8 ± 2.0	0.75	2.8 ± 1.9	3.7 ± 2.3	0.45	4.2 ± 2.7	2.6 ± 1.3	0.08
**Laboratory findings at d0**									
White blood cells (×10^9^ cell/L)	10,787 ± 4,029	10,738 ± 4,003	0.97	12,868 ± 4,819	10,209 ± 3,731	0.2	9,499 ± 3,208	12,193 ± 4,498	0.11
Neutrophils (×10^9^ cell/L)	4,792 [2,393–4,712]	3,587 [2,659–5,966]	0.26	6,419 [4,928–6,926]	3,510 [2,245–5,272]	0.06	3,728 [2,045–5,184]	4,928 [3,144–6,713]	0.21
Basophils (×10^9^ cell/L)	29 [28–43]	31 [22–54]	0.87	32 [29–55]	29 [20–51]	0.53	29 [21–45]	32 [27–58]	0.65
Eosinophils (×10^9^ cell/L)	39 [15–239]	83 [22–126]	0.47	96 [40–143]	32 [12–97]	0.22	28 [10–120]	40 [31–120]	0.37
Lymphocytes (×10^9^ cell/L)	4,963 ± 2,402	5,483 ± 3,959	0.62	4,799 ± 2,480	5,008 ± 2,451	0.87	4,585 ± 2,050	5,375 ± 2,776	0.44
Monocytes (×10^9^ cell/L)	1,040 ± 459	841 ± 239	0.15	1,303 ± 351	967 ± 466	0.15	913 ± 455	1,179 ± 4,441	0.17
C-reactive protein (mg/dL)	0.9 [0.04–9.7]	0.9 [0.04–12.6]	0.96	0.6 [0.2–1.6]	1.0 [0–9.7]	0.85	1.14 [0.04–4.11]	0.59 [0.08–9.70]	0.78

**Table 2:** LOS in hospital and BCS value at admission in association to demographic and clinical patient characteristics, laboratory findings and RSV load (mean log_10_ copies/ng hDNA). Continuous variables were presented as median [IQR] or mean ± SD, according to data distribution. n.e.^§^: statistic test not evaluable (frequency data equal to 0); n.e.^$^: data not evaluable (≤1 available data for row or column); *: data not available for all subjects; d0 coincides with patient’s admission to hospital; ∆: viral decay; significant correlations are highlighted in bold type.

	LOS in Hospital (days)			BCS at Admission		
	≤5 (n = 23)	>5 (n = 15)	*p* Value	Mild BCS (n = 30)	Moderate BCS (n = 8)	*p* Value
**RSV load**						
d0	4.46 ± 0.89	4.47 ± 1.04	0.97	4.47 ± 1.01	4.45 ± 0.63	0.96
d2	4.10 ± 1.04	4.29 ± 1.27	0.63	4.19 ± 1.01	4.17 ± 1.84	0.97
d4	3.01 ± 1.49	2.32 ± 1.48	0.32	2.79 ± 1.63	2.87 ± 0.94	0.92
d6	2.52 ± 094	2.10 ± 2.05	0.71	2.27 ± 1.81	1.97 ± 2.11	0.80
Peak	0.32 ± 1.20	0.17 ± 1.26	0.72	4.84 ± 0.79	4.63 ± 0.86	0.52
∆ d0–d2	4.76 ± 0.78	4.85 ± 0.85	0.73	0.25 ± 1.22	0.31 ± 1.29	0.91
∆ d0–d4	n.e.$	n.e.$	n.e.$	n.e.$	n.e.$	n.e.$
**Demographic characteristics**						
Age (month)	4.8 ± 3.2	2.6 ± 2.1	**0.03**	3.8 ± 2.8	4.6 ± 3.6	0.5
Sex (male)	10	9	0.5	14	5	0.7
Ethnicity (total available n *)	(n * = 21)	(n = 15)	n.e.§	(n * = 29)	(n * = 7)	n.e.§
Caucasian	17	11		23	5	
Asian	0	2		1	1	
African-American	4	2		5	1	
**Clinical characteristics**						
Premature birth (<37 WG)	5	1	0.22	6	0	n.e.$
Weight on admission (kg)	6.2 ± 2.0	5.4 ± 1.4	0.2	6.0 ± 1.8	5.6 ± 1.8	0.6
Breastfeeding	20	12	0.7	24	8	0.3
Duration of symptoms prior to d0 (days)	3.3 ± 1.9	3.9 ± 2.5	0.4	3.8 ± 2.3	2.9 ± 1.6	0.2
**Laboratory findings**						
White blood cells (×109 cell/L)	10,438 ± 3,851	11,356 ± 4,245	0.5	10,366 ± 3,523	12,441 ± 5,441	0.2
Neutrophils (×109 cell/L)	3,874 [2,659–4,952]	4,852 [1,898–6,499]	0.6	3,874 [2,626–4,928]	6,499 [2,850–8,400]	0.2
Basophils (×109 cell/L)	29 [22–43]	34 [29–61]	0.9	29 [22–43]	52 [38–58]	**0.04**
Eosinophils (×109 cell/L)	37 [15–116]	46 [29–232]	0.3	40 [18–143]	30 [15–126]	0.7
Lymphocytes (×109 cell/L)	5,199 ± 3,515	5,066 ± 1,944	0.9	5,129 ± 3,194	5,239 ± 2,293	0.9
Monocytes (×109 cell/L)	836 ± 322	1,203 ± 432	**0.01**	965 ± 346	981 ± 618	0.9
C-reactive protein (mg/dL)	0.90 [0.21–1.44]	0.80 [0.28–1.75]	0.6	0.85 [0.16–1.43]	2.06 [0.46–5.70]	0.2

**Table 3:** Relationship between patients’ demographic, clinical, and laboratory data and RSV load (at d0 time point and at peak value). * Spearman’s rho test; significant correlations are highlighted in bold type.

	RSV Load d0		Peak	
	r *	*p* Value	r *	*p* Value
**Demographic characteristics**				
Age (months)	−0.12	0.46	0.08	0.64
**Clinical characteristics**				
Weight (kg)	−0.26	0.12	−0.02	0.91
Duration of symptoms prior to d0 (days)	−0.003	0.98	0.002	0.99
**Laboratory findings**				
White blood cells (×10^9^ cell/L)	0.14	0.41	−0.35	**0.03**
Neutrophils (×10^9^ cell/L)	−0.06	0.72	−0.14	0.43
Basophils (×10^9^ cell/L)	−0.1	0.57	−0.23	0.17
Eosinophils (×10^9^ cell/L)	0.09	0.59	0.08	0.62
Lymphocytes (×10^9^ cell/L)	−0.16	0.37	−0.34	**0.04**
Monocytes (×10^9^ cell/L)	0.09	0.59	−0.02	0.91
C-reactive protein (mg/dL)	−0.25	0.14	−0.4	**0.01**
**Clinical outcomes**				
Duration of oxygen therapy	0.42	**0.04**	0.34	0.11
LOS in the hospital	0.11	0.49	0.09	0.61

## Data Availability

Data is available by contacting authors.

## Supplementary Materials

The following supporting information can be downloaded at: https://www.mdpi.com/article/10.3390/pathogens12050645/s1, Table S1. Studies with results supporting a direct relation between RSV load and bronchiolitis severity. Table S2. Studies finding an inverse correlation between RSV load and bronchiolitis severity. Table S3. Studies finding no correlation between RSV load and bronchiolitis severity.
