# Supplementary material for: Respiratory Syncytial Virus-Load Kinetics and Clinical Course of Acute Bronchiolitis in Hospitalized Infants: Interim Results and Review of the Literature
Source: Pathogens. 2023 Apr 27;12(5):645. doi: 10.3390/pathogens12050645 (PMC10221166; doi:10.3390/pathogens12050645)
Supplement: Supplementary file 1 [file pathogens-12-00645-s001.zip › pathogens-2346339-supplementary.pdf]

Supplementary materials

Reference	Study design and population, Specimen collection, Exclusion criteria	Sample size and number of cases	Outcome – measure of interest	Results (p value)
[8]	Prospective study on hospitalized children (10 d – 4 y, median age 4 mo) with RSV ARI, in a subgroup of 19 infants < 11 mo NW at admission and every 1-3 d throughout hospitalization with RSV culture (log_{10} $TCID_{50}$/mL), **No exclusion criteria**	59 patients,<br>19/59 infants with NW,<br>17/19 with pneumonia ± bronchiolitis,<br>2/19 with bronchiolitis	Presence of pulmonary consolidation,<br>Hypoxia,<br>LOS	Significant correlation between greater quantities of RSV and pulmonary consolidation ($p < 0.025$), no significant correlation between greater quantities of RSV and hypoxia and/or prolonged LOS ($p > 0.05$)
[9]	Prospective study on hospitalized < 2 years children with RSV infection, nasal aspirate at admission with RSV culture (log_{10} PFU/mL)<br>**Exclusion criteria**: BPD, hemodynamically significant CHD, ID,<br>SCS treatment within the 3 weeks before admission, Ribavirin treatment, RSV immunoglobulin or RSV-specific Ab treatment within the 3 months before enrollment, need for MV for apnea	39 patients,<br>15/39 severe bronchiolitis<br>(= need for MV during hospitalization),<br>24/39 non-severe	Disease severity<br>(= need for MV during hospitalization)	Significant correlation between higher nasal RSV quantities and severe bronchiolitis<br>($p = 0.022$)
[11]	Prospective observational study on previously healthy children < 2 years hospitalized for RSV bronchiolitis, nasal aspirate at admission with RSV culture (log_{10} PFU/mL)<br>**Exclusion criteria**: BPD, ID, hemodynamically significant CHD,<br>SCS treatment in the preceding month, positive blood cultures	141 patients	LOS,<br>Need for PICU,<br>need for MV	Significant correlation between higher nasal RSV quantity and longer LOS ($p = 0.008$; a 1 log PFU/mL higher RSV load predicted 0.8 days longer LOS),<br>need for PICU ($p = 0.0049$),<br>need for MV ($p = 0.0048$)
[12]	Prospective study on previously healthy infants < 1 year hospitalized for RSV bronchiolitis, NPA (once for patient) with RSV load by RT-PCR (RSV-RNA transcript/µL)<br>**Exclusion criteria**: BPD, Hemodynamically significant CHD, ID, SCS treatment within the 3 weeks before admission, Ribavirin treatment, RSV-specific Ab preparations within the 3 months before enrollment	81 patients,<br>28/81 with risk factors for severe disease (prematurity and/or LBW),<br>53/81 without risk factors	Severe disease intended as:<br>- RR > 60 bpm,<br>- LOS > 7 days,<br>- Need for PICU,<br>- Need for MV	Significant correlation between higher RSV load and severe bronchiolitis in both patients with risk factors ($p = 0.024$) and without risk factors ($p = 0.027$)
[13]	Prospective observational study on previously healthy infants < 6 mo outpatients with first RTI, NPA at enrollment with viral load expressed by PCR-CT values (inverse linear relationship between viral load and CT-values)<br>**Exclusion criteria**: NA	82 infants,<br>30/82 infants with RSV infection,<br>11/30 infants with RSV as sole pathogen	Specified disease severity score	Significant correlation between RSV viral load and disease severity score value in infants with RSV infection ($p = 0.003$) even when RSV as sole pathogen ($p = 0.02$)
[14]	Prospective monocentric study on previously healthy children ≤ 2 years	219 patients,	Hospital admission,	Significant correlation between higher RSV load

	with RSV infection, NPA or ET aspirate at admission and daily until discharge, RSV load ($\log_{10}$ PFU/mL)<br>**Exclusion criteria**: BPD, hemodynamically significant CHD, ID, SCS treatment within the preceding month, positive blood cultures	192/219 hospitalized<br>218/219 samples on day 1,<br>102/219 samples on day 2,<br>41/219 samples on day 3	LOS,<br>Need for PICU,<br>Need for MV	and prolonged LOS ($p < 0.001$; RSV load increase of 1 log PFU/mL predicted 1 additional day in LOS), between higher RSV load on day 3<br>and need for PICU ($p = 0.009$), between reduced clearance (Δ viral load d 1-3) and prolonged LOS<br>($p = 0.035$)
[16]	Prospective study on children < 3 years (median age 2.2 mo) hospitalized for RSV bronchiolitis, NPW at admission, RSV load (log copy number of RSV-RNA/mL)<br>**Exclusion criteria**: Underlying chronic condition (prematurity, BPD, CF, CHD, ID) and recurrent (more than one) wheezing episodes	132 patients	Specified disease severity score,<br>LOS	Significant correlation between higher RSV load and higher value of severity score ($p = 0.024$) and between higher RSV load and prolonged LOS<br>($p = 0.038$)
[17]	Prospective study on previously healthy children ≤ 2 years hospitalized for bronchiolitis, NPA at admission, day 3, day 5, day 7, RSV load ($\log_{10}$ copy number of RSV-RNA/mL)<br>**Exclusion criteria**: Recurrent wheezing, Ribavirin or SCS treatment in the preceding two weeks	60 patients,<br>40/60 with RSV bronchiolitis<br>(age < 1 year),<br>18/40 severe bronchiolitis,<br>18/40 moderate bronchiolitis,<br>4/40 mild bronchiolitis	Specified disease severity score	Significant correlation between higher RSV load and higher disease severity score value ($p < 0.001$), significant differences in the mean RSV load between the mild group and the severe group ($p = 0.014$)
[18]	Prospective multicenter study on children < 2 years hospitalized for bronchiolitis, NPA at admission, RSV load expressed by<br>PCR-CT values (inverse linear relationship between viral<br>load and CT-values)<br>**Exclusion criteria**: Enrollment in previous studies, Enrollment > 48 hours since hospital admission	2615 patients,<br>1764/2615 RSV infection,<br>587/1764 low RSV genomic load,<br>598/1764 medium load,<br>579/1764 high load,<br>1223 patients with a stricter definition of bronchiolitis<br>(age ≤ 1 year, GA ≥ 37 weeks)	LOS,<br>need for PICU	Significant correlation between higher RSV genomic load and risk of LOS ≥ 3 days (OR 1.58, $p < 0.001$), even in the subgroup with a stricter definition (OR 1.46, $p = 0.01$), significant correlation between higher RSV genomic load and PICU (OR 1.43, $p = 0.03$), without correlation in the subgroup without a stricter definition ($p > 0.05$)
[19]	Double-blinded RCT (inhaled racemic adrenaline vs. saline) on infant < 1 year hospitalized for bronchiolitis with specified severity score values ≥ 4 (moderate-severe illness), NPA at admission with RSV load expressed by<br>PCR-CT values (inverse linear relationship between viral<br>load and CT-values)<br>**Exclusion criteria**: presence of any serious cardiac, immunologic, neurologic, oncologic, pulmonary disease other than bronchiolitis,<br>>1 previous episode of obstructive airway disease, symptoms of disease of	404 patients,<br>363/404 patients with NPA,<br>300/363 RSV infection,<br>200/363 high RSV load,<br>89/363 RSV single infection	LOS,<br>need for oxygen therapy,<br>need for ventilatory support	Significant association between higher RSV load and prolonged LOS ($p = 0.042$),<br>need for oxygen therapy ($p = 0.006$) or ventilatory support ($p = 0.021$)

	the lower airway (e.g. coughing) for >4 weeks, An CS treatment in the preceding 4 weeks			
[22]	Observational analytical cross-sectional study on infants < 1 year hospitalized for RSV LRTI, NPA < 3 d or ≥ 3 d from illness with RSV genomic load expressed by PCR-CT values (inverse linear relationship between viral load and CT-values)<br>**Exclusion criteria**: Prematurity, ID, documented chronic lung or heart disease, possible bacterial infection	86 infants with RSV LRTI<br>49/86 moderate clinical score disease severity values,<br>37/86 severe clinical score disease severity values	Specified clinical score disease severity	Significant correlation between higher RSV genomic loads and severe RSV LRTI in later LRTI stage (NPA ≥ 3 d; p = 0.04)
[25]	Prospective study on infants 0-6 mo hospitalized for RSV±RV bronchiolitis, NPA at admission with RSV load expressed by PCR-CT values (inverse linear relationship between viral load and CT-values)<br>**Exclusion criteria**: Recent (≤ 30 d before current infection) or current SCS treatment, Recent (≤ 15 d before current infection) or current antibiotic treatment, Previous wheezing crisis, CHD, BPD	70 patients,<br>61/70 infants RSV infection,<br>49/61 sole-RSV infection,<br>12/61 RSV+RV infection	LOS,<br>ICU stay,<br>Oxygen therapy duration,<br>Respiratory support duration	In patients with sole-RSV infection, higher RSV load is a risk factor for prolonged LOS<br>(OR 3.23, p = 0.012), prolonged ICU stay<br>(OR 2.03, p = 0.036), prolonged oxygen therapy duration (OR 2.96, p = 0.011), prolonged respiratory support duration<br>(OR 1.84, p = 0.021); in patients with RSV+RV infection, a higher RSV load is a risk factor for prolonged oxygen therapy duration (OR 4.78, p = 0.017)

Table S1. Studies with results supporting a direct relation between RSV load and bronchiolitis severity.

Note: Ab = antibodies, ARI = acute respiratory infection, BPD = bronchopulmonary dysplasia, bpm = breaths per minute, CF = cystic fibrosis, CHD = congenital heart disease, CPAP = Continuous Positive Airways Pressure, CT = cycle threshold, d = days, ED = Emergency Department, ET = endotracheal tube, GW = General Ward, ID = immunodeficiency, IVIG = intravenous immunoglobulin, LBW = low birth weight, LOS = length of stay, LRTI = low respiratory tract infection, mo = months, MV = mechanical ventilation, NA = not available, NPA = nasopharyngeal aspirate, NPW = nasopharyngeal wash, NW = nasal wash, PFU = plaque-forming units, PICU = Pediatric Intensive Care Unit, RTI = respiratory tract infection, RT-PCR = Real Time Polymerase Chain Reaction, SCS = systemic corticosteroids, RR = respiratory rate, RSV = Respiratory Syncytial Virus, RV = Rhinovirus, $SatO_2$ = oxygen saturation, $TCID_{50}$ = 50% tissue culture infectious dose, URTI = upper respiratory tract infection, w = weeks, y = years.

Reference	Study design and population, Specimen collection, Exclusion criteria	Sample size and number of cases	Outcome – measure of interest	Results (p value)
[23]	Prospective cross-sectional study on children ≤ 2 years with diagnosis of bronchiolitis in ED, NW at enrollment with copy numbers measurement of 5 different RSV genes (NS1, NS2, N, G and F)<br>**No exclusion criteria**	112 patients,<br>79/112 with RSV infection,<br>41/79 hospitalized patients,<br>38/79 outpatients	Hospitalization,<br>$SatO_2$	Significant correlation between higher RSV gene copy numbers (except F) and non-hospitalization (p = 0.01 – 0.04);<br>significant correlation between higher RSV gene NS1, NS2, N copy numbers and higher $SatO_2$ (p = 0.03)
[24]	Prospective study on children 2 w – 2 y and weight > 2 Kg (also with non-respiratory comorbidities, e.g. prematurity) hospitalized with PCR-confirmed viral bronchiolitis, NPA up to twice daily during hospitalization with RSV load (RSV-RNA copies/mL)<br>**Exclusion criteria:** any local or systemic factor that could influence the safety of nasal sampling (e.g., bleeding disorders), presence of nasal cannulae in both nostrils, palliative care, respiratory comorbidity	55 patients,<br>30/55 patients with RSV,<br>12/30 severe (PICU),<br>18/30 moderate (GW)	Severity disease defined by<br>the type of hospitalization<br>(PICU or GW)	Significantly higher RSV load in moderate than in severe cases (p = 0.001), with a significant decreasing in viral load in severe RSV cases relative to moderate ones (p = 0.001)
[7]	Prospective study on healthy children < 2 years hospitalized or evaluated in outpatient setting for RSV infection, nasal swab only at admission in outpatients, once a day during hospitalization, with RSV load<br>(log_{10} copy number of RSV-RNA/mL)<br>**Exclusion criteria**: duration of illness >7 days, prematurity, underlying medical conditions (i.e. CHD, BPD, ID), use of immunomodulatory drugs (including SCS) > 5 days within 2 weeks of presentation, previous<br>history of wheezing or hospitalization for RSV bronchiolitis	150 children with RSV infection,<br>111/150 hospitalized<br>39/150 outpatients,	Specified clinical severity score,<br>Hospitalization,<br>Need for oxygen therapy	Significant correlation between higher RSV load and outpatients (p = 0.003), lower clinical score value in outpatients (p = 0.0002), lower need of oxygen therapy in hospitalized patients (p = 0.04)

Table S2. Studies finding an inverse correlation between RSV load and bronchiolitis severity.

Reference	Study design and population, Specimen collection, Exclusion criteria	Sample size and number of cases	Outcome – measure of interest	Results (p value)
[10]	Multicenter double-blind placebo-controlled RCT on children ≤ 2 years hospitalized with RSV bronchiolitis, admission and subsequently daily NW or ET aspirate with RSV culture (log_{10} PFU/mL)<br>**Exclusion criteria**: ID, CF, reactive airways disease or asthma, poorly controlled CHD, renal failure, ventilator dependency at the enrollment,<br>life expectancy < 6 mo, apnea without evidence of LRTI,<br>IVIG therapy in the preceding 2 months, Ribavirin in the preceding month	77 patients,<br>33/77 healthy infants,<br>18/77 infants with CHD ± BPD ± prematurity (< 32 w),<br>11/77 infants with BPD ± prematurity,<br>15/77 infants with prematurity	Specified respiratory illness score	No correlation between severity of illness judged by respiratory illness score and RSV titer (p > 0.05)
[15]	Cross-sectional study on children ≤ 2 years hospitalized for LRTI,<br>NPA at admission with RSV load expressed by PCR-CT values (inverse linear relationship between viral load and CT-values)<br>**Exclusion criteria**: NA	120 patients,<br>98/120 viral positive NPA,<br>58/98 with RSV infection,<br>27/98 with RV infection,<br>11/98 RSV+RV infection	LOS	Trend, although not significant, in correlation between RSV CT-value and LOS (p = 0.05)
[20]	Cross-sectional study on infants ≤ 1 year hospitalized for bronchiolitis,<br>NPW at admission with RSV load (copies/mL)<br>**Exclusion criteria**: BPD, CHD, CF, bronchiolitis obliterans, Bordetella pertussis infection, previous macrolides treatment	110 patients,<br>56/110 samples analyzed with RSV load by quantitative RT-PCR	Duration of wheezing,<br>LOS<br>Duration of oxygen therapy	No correlation between RSV load and duration of wheezing (p > 0.05),<br>LOS (p > 0.05),<br>duration of oxygen therapy<br>(p > 0.05)
[21]	Prospective study on children (age not specified) hospitalized for LRTI (pneumonia, bronchitis, bronchiolitis), NPA at admission with RSV load (copies/mL)<br>**No exclusion criteria**	387 patients,<br>160/387 patients with bronchiolitis,<br>RSV 1st pathogen in bronchiolitis (data about frequency NA)	Specified criteria for severity disease definition	No correlation between RSV load and severity disease defined by specified clinical signs (p > 0.05)
[26]	Prospective study on children < 2 years hospitalized for RSV bronchiolitis,<br>NP swab at admission and daily during hospitalization, then weekly until no more RSV detection, with RSV viral load (log_{10} RNA copies/mL)<br>**Exclusion criteria**: Use of Palivizumab, drop-out at follow-up	38 patients,<br>247 samples	Need for PICU,<br>Need for intubation,<br>LOS	No significant differences in RSV load and<br>need for PICU (p > 0.05),<br>nor for intubation (p > 0.05), nor prolonged LOS (p > 0.05)

Table S3. Studies finding no correlation between RSV load and bronchiolitis severity.

References

7. Garcia-Mauriño, C.; Moore-Clingenpeel, M. Viral Load Dynamics and Clinical Disease Severity in Infants With Respiratory Syncytial Virus Infection. *J Infect Dis* **2019**, *219(8)*, 1207-1215.

8. Hall, C.B.; Douglas, R.G. Respiratory syncytial virus infections in infants: quantitation and duration of shedding. *J Pediatr* **1976**, *89(1)*, 11-15.

9. Buckingham, S.C.; Bush, A.J. Nasal quantity of respiratory syncytial virus correlates with disease severity in hospitalized infants. *Pediatr Infect Dis J* **2000**, *19(2)*, 113-117.

10. Wright, P.F.; Gruber, W.C. Illness severity, viral shedding, and antibody responses in infants hospitalized with bronchiolitis caused by respiratory syncytial virus. *J Infect Dis* **2002**, *185(8)*, 1011-1018.

11. DeVincenzo, J.P.; El Saleeby, C.M. Respiratory syncytial virus load predicts disease severity in previously healthy infants. *J Infect Dis* **2005**, *191(11)*, 1861-1868.

12. Fodha, I.; Vabret, A. Respiratory syncytial virus infections in hospitalized infants: association between viral load, virus subgroup, and disease severity. *J Med Virol* **2007**, *79(12)*, 1951-1958.

13. Houben, M.L.; Coenjaerts, F.E. Disease severity and viral load are correlated in infants with primary respiratory syncytial virus infection in the community. *J Med Virol* **2010**, *82(7)*, 1266-1271.

14. El Saleeby, C.M.; Bush, A.J. Respiratory syncytial virus load, viral dynamics, and disease severity in previously healthy naturally infected children. *J Infect Dis* **2011**, *204(7)*, 996-1002.

15. Van Leeuwen, J.C.; Goossens, L.K. Equal virulence of rhinovirus and respiratory syncytial virus in infants hospitalized for lower respiratory tract infection. *Pediatr Infect Dis J* **2012**, *31(1)*, 84-86.

16. Scagnolari, C.; Midulla, F. Evaluation of viral load in infants hospitalized with bronchiolitis caused by respiratory syncytial virus. *Med Microbiol Immunol* **2012**, *201(3)*, 311-317.

17. Zhou, L.; Xiao, Q. The impact of viral dynamics on the clinical severity of infants with respiratory syncytial virus bronchiolitis. *J Med Virol* **2015**, *87(8)*, 1276-1284.

18. Hasegawa, K.; Jartti, T. Respiratory syncytial virus genomic load and disease severity among children hospitalized with bronchiolitis: multicenter cohort studies in the United States and Finland. *J Infect Dis* **2015**, *211(10)*, 1550-1559.

19. Skjerven, H.O.; Megremis, S. Virus Type and Genomic Load in Acute Bronchiolitis: Severity and Treatment Response With Inhaled Adrenaline. *J Infect Dis* **2016**, *213(6)*, 915-921.

20. Souza, A.P.; Leitão, L.A. Lack of association between viral load and severity of acute bronchiolitis in infants. *J Bras Pneumol* **2016**, *42(4)*, 261-265.

21. Yan, X.L.; Li, Y.N. Clinical characteristics and viral load of respiratory syncytial virus and human metapneumovirus in children hospitalized for acute lower respiratory tract infection. *J Med Virol* **2017,** *89(4)*, 589-597.

22. Espinosa, Y.; San Martín, C. Genomic Loads and Genotypes of Respiratory Syncytial Virus: Viral Factors during Lower Respiratory Tract Infection in Chilean Hospitalized Infants. Int J Mol Sci. 2017 Mar 21;18(3):654. doi: 10.3390/ijms18030654. PMID: 28335547; PMCID: PMC5372666.

23. Piedra, F.A.; Mei, M. The interdependencies of viral load, the innate immune response, and clinical outcome in children presenting to the emergency department with respiratory syncytial virus-associated bronchiolitis. PLoS One. 2017 Mar 7;12(3):e0172953. doi: 10.1371/journal.pone.0172953. PMID: 28267794; PMCID: PMC5340370.

24. Thwaites, R.S.; Coates, M. Reduced Nasal Viral Load and IFN Responses in Infants with Respiratory Syncytial Virus Bronchiolitis and Respiratory Failure. *Am J Respir Crit Care Med* **2018**, *198(8)*, 1074-1084.

25. De Paulis, M.; Oliveira, D.B.L. The importance of viral load in the severity of acute bronchiolitis in hospitalized infants. *Clinics (Sao Paulo)* **2021**, *76*, e3192.

26. Watanabe, R.A.S.; Cruz, J.S. Respiratory syncytial virus: viral load, viral decay, and disease progression in children with bronchiolitis. *Braz J Microbiol* **2022**, *53(3)*, 1241-1247.

Disclaimer/Publisher's Note: The statements, opinions and data contained in all publications are solely those of the individual author(s) and contributor(s) and not of MDPI and/or the editor(s). MDPI and/or the editor(s) disclaim responsibility for any injury to people or property resulting from any ideas, methods, instructions or products referred to in the content.
